# Longitudinal Three-Year Associations of Dietary Fruit and Vegetable Intake with Serum hs-C-Reactive Protein in Adults with and without Type 1 Diabetes

**DOI:** 10.3390/nu16132058

**Published:** 2024-06-28

**Authors:** Macy M. Helm, Arpita Basu, Leigh Ann Richardson, Lung-Chang Chien, Kenneth Izuora, Amy C. Alman, Janet K. Snell-Bergeon

**Affiliations:** 1 Department of Kinesiology and Nutrition Sciences, University of Nevada at Las Vegas, Las Vegas, NV 89154, USA; helmm1@unlv.nevada.edu (M.M.H.); richal7@unlv.nevada.edu (L.A.R.); 2 Department of Epidemiology and Biostatistics, University of Nevada at Las Vegas, Las Vegas, NV 89154, USA; lung-chang.chien@unlv.edu; 3 Section of Endocrinology, School of Medicine, University of Nevada at Las Vegas, Las Vegas, NV 89154, USA; kenneth.izuora@unlv.edu; 4 College of Public Health, University of South Florida, Tampa, FL 33612, USA; aalman@usf.edu; 5 Barbara Davis Center for Diabetes, University of Colorado, Anschutz Medical Campus, Aurora, CO 80045, USA; janet.snell-bergeon@cuanschutz.edu

**Keywords:** C-reactive protein, dietary approaches to stop hypertension dietary score, alternative healthy eating index dietary score, blueberries

## Abstract

High-sensitivity C-reactive protein (hs-CRP) is a widely used clinical biomarker of systemic inflammation, implicated in many chronic conditions, including type 1 diabetes (T1D). Despite the increasing emphasis on dietary intake as a modifiable risk factor for systemic inflammation, the association of hs-CRP with fruit and vegetable consumption is relatively underexplored in T1D. To address this gap, we investigated the longitudinal associations of dietary pattern-derived fruit and vegetable scores with hs-CRP in adults with and without T1D. Additionally, we examined the impact of berry consumption as a distinct food group. Data were collected in the Coronary Artery Calcification in Type 1 Diabetes study over two visits that were three years apart. At each visit, participants completed a food frequency questionnaire, and hs-CRP was measured using a particle-enhanced immunonephelometric assay. Mixed effect models were used to examine the three-year association of fruit and vegetable scores with hs-CRP. Adjusted models found a significant inverse association between blueberry intake and hs-CRP in the nondiabetic (non-DM) group. Dietary Approaches to Stop Hypertension- and Alternative Healthy Eating Index-derived vegetable scores were also inversely associated with hs-CRP in the non-DM group (all *p*-values ≤ 0.05). Conversely, no significant associations were observed in the T1D group. In conclusion, dietary pattern-derived vegetable scores are inversely associated with hs-CRP in non-DM adults. Nonetheless, in T1D, chronic hyperglycemia and related metabolic abnormalities may override the cardioprotective features of these food groups at habitually consumed servings.

## 1. Introduction

Globally, noncommunicable diseases continue to be the leading cause of mortality, resulting in approximately 41 million deaths annually [1]. Within this classification, cardiovascular disease and diabetes are two of the primary conditions [1,2,3,4]. Despite their varied pathophysiological mechanisms, systemic inflammation plays a consistent role in the etiology of these chronic diseases [5]. Type 1 diabetes (T1D) has been associated with elevated systemic inflammation and increased risk of coronary heart disease [6]. Meta-analyses of inflammatory markers in T1D have reported significantly elevated levels of interleukin 1-beta [7], interleukin-6 (IL-6) [8], and tumor necrosis factor alpha (TNF-α) [9]. In T1D, these elevated inflammatory markers contribute to insulitis, beta cell death, and suppressed beta cell regeneration [10,11]. Increased inflammation during diabetes increases the risk of diabetes-related macro- and microvascular complications [12].

C-reactive protein (CRP) is an important inflammatory marker that elevates in T1D [13], increases the risk of all-cause mortality in diabetes [14,15], and predicts cardiovascular events in asymptomatic populations [16] and those with poorly controlled diabetes [17]. Proinflammatory cytokines such as IL-6 and TNF-α stimulate the production of CRP by hepatocytes [18]. Considering the elevated inflammation in T1D and the role these markers have in activating CRP and subsequently increasing the risk of complications, research continues to explore anti-inflammatory therapies, including dietary supplementation, adjuvant therapies, and eating styles [19,20,21,22,23]. As a modifiable behavior, dietary intake is an important mechanism to address inflammation.

Several epidemiological studies have shown that plant-based foods and dietary patterns, especially those rich in fruits and vegetables, are associated with less chronic inflammation, including lower circulating CRP levels as measured by high-sensitivity (hs-) and standard assay [24,25,26,27]. These associations are mainly attributed to anti-inflammatory properties from various dietary bioactive compounds, including fiber and polyphenols. In a meta-analysis of 17 observational studies pooling data from healthy adults and those with type 2 diabetes, adherence to a vegetarian diet for a minimum of two years was associated with significantly lower circulating levels of hs-CRP [28]. In adults with type 2 diabetes, better diet quality (i.e., more plant-based foods and less processed foods) was significantly associated with lower standard assay CRP concentrations in males and females across five ethnic groups [29]. In middle-aged and older adults, consuming a greater number of diverse fruits and vegetables (e.g., apples, bananas, peaches, nectarine, papaya, mangoes, prunes, pineapples, lettuce, spinach, carrots, peas, corn, peppers, garlic, and zucchini) on a monthly basis has been associated with lower concentrations of hs-CRP [30]. Vegetable intake has also been associated with reduced odds of elevated standard assay CRP in healthy middle-aged adults over a 12-year period [31]. A longitudinal analysis of healthy middle-aged adults has reported inverse associations between overall diet quality and serum CRP levels [32]. Across a pooled analysis of cohort, case–control, cross-sectional, and randomized controlled studies (94% conducted in adults with and without cardiometabolic risk factors), fruit and vegetable intake, independently and combined, were associated with reduced levels of hs-CRP [33].

In randomized controlled trials, adhering to a Mediterranean, healthy, and/or anti-inflammatory diet decreased circulating CRP (hs- and standard assay) in adults with type 2 diabetes compared to a usual or habitual diet [34]. The Mediterranean Diet also reduced circulating CRP (hs- and standard assay) in healthy adults [35] and in adults with cardiometabolic risk factors [36]. While these findings represent the effectiveness of a dietary pattern rich in fruits and vegetables, randomized controlled trials administering single-berry fruits have also shown reductions in CRP. In a meta-analysis of 17 studies, pooled analysis across healthy adults and those with cardiometabolic conditions reported a significant reduction in CRP (hs- and standard assay) from consuming grape products [37]. A meta-analysis of multiple single-berry interventions in clinical trials reported significant reductions in CRP (hs- and standard assay) across healthy adults, those with metabolic syndrome and type 2 diabetes, and those with cardiovascular risk factors [38]. We have also previously demonstrated that raspberry intake reduces IL-6 and TNF-α in individuals with type 2 diabetes four hours and four weeks after consumption [39].

Considering the relationship between dietary intake and inflammation, three dietary patterns have been studied for their associations or effects on inflammation: Mediterranean Diet [34,35,36], Dietary Approaches to Stop Hypertension (DASH) [40], and the Alternative Healthy Eating Index (AHEI) [41]. These dietary patterns were also associated with significantly reduced risk of all-cause and cardiovascular mortality [42,43]. High adherence to these patterns indicates a high intake of fruits and vegetables, among other plant-based foods, which can be quantified using scoring metrics. Using these dietary indices to understand the associations between dietary intake and health outcomes strengthens findings as the indices each have common characteristics (e.g., emphasis on fruits and vegetables) that create consistency across findings.

While most of the observational studies and clinical trials that included cardiometabolic conditions focused on type 2 diabetes, few studies have also investigated associations between diet and serum hs-CRP in T1D. In adults with T1D, reduced fiber intake was associated with greater low-grade inflammation, measured as a combination of hs-CRP, IL-6, and TNF-α [44]. Hs-CRP levels have also been inversely correlated to diet quality scores in adults with T1D [45,46]. Despite these few studies assessing anti-inflammatory features of dietary patterns, the associations of fruit and vegetable intake, as well as berries, with circulating hs-CRP in T1D are less understood.

Thus, the primary objective of this study is to investigate the associations of dietary fruit and vegetable scores derived from the Mediterranean Diet, DASH, and AHEI patterns with circulating hs-CRP. These scores will be examined with and without berries to better understand associations with this anti-inflammatory single-fruit group. These associations were examined in an established cohort of adults with and without T1D in the Coronary Artery Calcification in Type 1 Diabetes (CACTI) study with the original goal to examine the progression of coronary artery disease and its determinants (including dietary) in adults with and without T1D.

## 2. Methods

### 2.1. Participants

The CACTI study is an ongoing prospective cohort study evaluating coronary artery calcification progression among individuals with and without T1D [47]. The inclusion and exclusion criteria have been previously described [48]. Briefly, participants were between 19 and 56 years old with no known history of cardiovascular disease: 652 with T1D and 764 nondiabetic controls (non-DM). Participants with T1D were all on insulin therapy within a year of diagnosis and were diagnosed prior to age 30 or had a clinical course consistent with T1D. The non-DM participants had fasting blood glucose < 126 mg/dL. Exclusion criteria included missing dietary information. In addition, women or men who reported a daily intake of ≥3500 kcal or ≥4000 kcal, respectively, were excluded using Willett’s criteria [49]. Similarly, women or men who reported a daily intake of <500 kcal or <800 kcal, respectively, were excluded [49].

**Figure 1:**
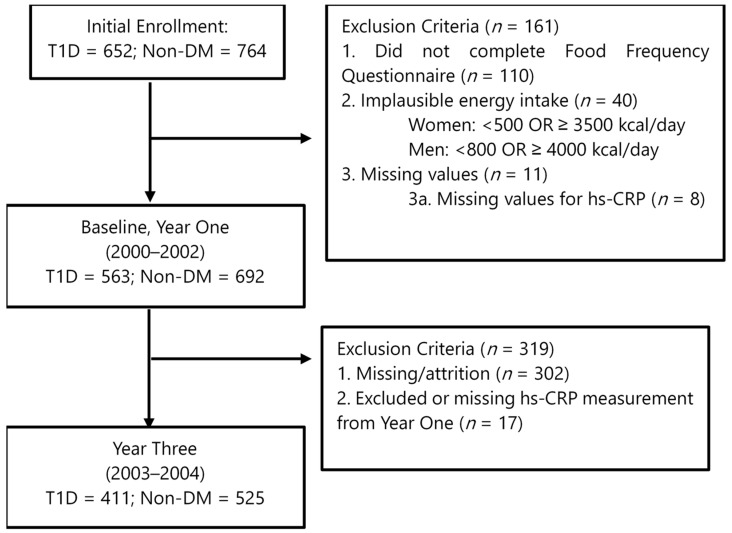
Flowchart of Coronary Artery Calcification in Type 1 Diabetes study design.

Data were collected over a 3-year period during two visits: baseline (2000–2002) and year three (2003–2004), see Figure 1. The study was approved by the Colorado Multiple Institutional Review Board.

### 2.2. Dietary Intakes and Pattern Scores

To measure dietary intake, a validated 126-item food frequency questionnaire was administered [50]. This tool has established reproducibility and validity to assess dietary patterns when used with adults [51,52].

Fruit and vegetable intakes were assessed by calculating dietary scores according to Mediterranean-Style Dietary Pattern Score (MSDPS), DASH, and AHEI. These scores specifically calculated the fruit and vegetable components and were calculated with and without berries.

The MSDPS has 13 components based on the Mediterranean Diet pyramid, with scores determined based on intake congruence with the recommended number of daily or weekly servings and a penalty for overconsumption [53]. Scores range from 0 to 10, representing a percentage of the recommended intake consumed [53]. DASH diet scores were calculated by classifying participants’ intake into quintiles and assigning 1–5 points corresponding to the quintile [54]. Scores for fruit and vegetable intake range from 2 to 10. AHEI diet scores were calculated by assigning 0 to 10 points to the intake of fruits and vegetables based on an a priori dietary index associated with decreased chronic disease risk [55].

### 2.3. Study Measurements

Standardized questionnaires were administered to collect demographics, smoking status, and physical activity, as previously described [47]. Illness status was assessed by asking if the patient had been recently ill with cold/flu symptoms or fever. Height and weight were collected through physical examination to calculate body mass index (BMI) [47]. Similarly, systolic blood pressure was measured during physical examination [47]. Hemoglobin A1c (HbA_1c_) was measured after an overnight fast using high-performance liquid chromatography [47].

### 2.4. Measurement of hs-CRP

High-sensitivity C-reactive protein (hs-CRP) was measured from blood collected from participants after a 12 h (overnight) fast, which has been previously described [56]. Briefly, blood was collected, centrifuged, and separated plasma was stored until ready for assay. The hs-CRP measurements were conducted using a particle-enhanced immunonephelometric assay. Hs-CRP concentrations > 3 mg/L were considered elevated [57].

### 2.5. Statistical Analysis

The primary objective of this analysis was to determine any significant association of dietary pattern-derived fruit and vegetable scores, calculated with and without berries, with circulating hs-CRP in a pooled sample and stratified by diabetes. Baseline characteristics were compared to year three values within respective diabetes statuses (i.e., non-DM at baseline compared to non-DM at year three). Comparisons of the two visits were examined using an independent samples test for continuous variables and the chi-square test was applied for categorical variables. For non-normally distributed continuous data, the Wilcoxon rank-sum test was applied with Monte Carlo estimation for *p*-values. Because the CACTI study design collected repeated measures on the same individuals over multiple visits, the general linear mixed model (continuous hs-CRP) and the generalized linear mixed model with a logit function (dichotomous hs-CRP) were applied to examine the three-year association of consumption of berries, fruit, and vegetable scores (independently) with hs-CRP, analyzed separately, after adjusted for relevant covariates in the models as follows: model 1—age, sex, calories, visit, and diabetes status (for pooled analysis); and model 2—all covariates in model 1, systolic blood pressure, body mass index (BMI), smoking status (never smoke, current smoker, and former smoker), and patient illness status (yes/no recently ill with cold/flu symptoms or fever). Patient illness status was included in the model to account for the potential effect of acute infection on hs-CRP values. [58] In all mixed-effect models, all covariates previously listed were fixed effects, and the repeated visits were the only random effect. The unstructured correlations (UNR) covariance structure was applied to the R matrix to control for temporal autocorrelation between the two visits. Additionally, the data for hs-CRP were right-skewed and log-transformed to increase precision in the general linear mixed model. Due to the log transformation, estimated coefficients ($\hat{\beta }$) were transformed using the equation (exp($\hat{\beta }$) − 1) × 100% to interpret the percentage change (%Change) in hs-CRP. The log-transformed results have the following interpretation: (1) Compared to those who did not eat any berries, those who consumed total berries, strawberries, or blueberries are associated with a/n X% increase or decrease in hs-CRP. (2) Compared to those who did not eat any berries, the MSDPS, DASH, or AHEI-derived fruit (with or without berries) or vegetable score is associated with a/n X% increase or decrease in hs-CRP. Additionally, hs-CRP was dichotomized as either >3 or ≤3 mg/dL because of the increased risk of cardiometabolic dysfunction with hs-CRP above 3 [57]. The results are expressed as an odds ratio and have the following interpretation: eating (total berries, strawberries, or blueberries) is associated with an increased (or decreased) odds of having a hs-CRP level above 3 mg/dL. Statistical analyses were performed using SAS v9.4 (SAS Institute Inc., Cary, NC, USA). A two-sided alpha level of ≤0.05 was used to define statistical significance.

## 3. Results

Table 1 shows the characteristic differences between baseline and year three for the non-DM and T1D participants. No significant differences were noted in strawberry and blueberry consumption between the time periods for either group. In the non-DM group, there was a significant increase in total vegetable consumption from baseline to year three (*p*-value = 0.0415). The T1D group did not have any significant differences in the fruit and vegetable scores from baseline to year three.

After adjusting for covariates, we found a significant inverse association between blueberry intake and serum hs-CRP in the non-DM group (Table 2). In the non-DM analysis, for every one-serving increase in blueberry intake, there was a significant −8.29% (95% CI = −14.50, −1.64; *p*-value = 0.0155) decrease in serum hs-CRP over three years. No significant associations were observed in the T1D group from total berries, blueberry, and strawberry intake. The calculated odds ratios of total berries, strawberry, and blueberry intake with the likelihood of clinically elevated hs-CRP (>3 mg/dL) were not significant in the pooled and stratified analyses (Table 3).

In Table 4, fruit and vegetable intake were analyzed using MSDPS, DASH, and AHEI-based indices. After adjusting for age, sex, calories, visit, and diabetes status (model 1), we found a significant inverse association between DASH-derived vegetable score and serum hs-CRP in the non-DM group. For every one-point increase in the DASH vegetable score, there was a significant −3.69% (95% CI = −6.69, −0.59; *p*-value = 0.0202) decrease in serum hs-CRP over the three-year period. Further adjustment of systolic blood pressure, BMI, smoking status, and patient illness status (model 2) yielded a persistently significant inverse association, where for every one-point increase in the DASH-derived vegetable score, there was a significant −3.22% (95% CI = −6.08, −0.28; *p*-value = 0.0321) decrease in serum hs-CRP over the three-year period. Model 1, adjusting for the same factors as above, for AHEI-derived vegetable score showed borderline significance (*p*-value = 0.0525) in the non-DM group. The association in the non-DM group was significant in the further adjusted model 2, where for every one-point increase in the AHEI-derived vegetable score, there was a significant −1.45% (95% CI = −2.88, 0.00; *p*-value = 0.0504) decrease in serum hs-CRP over the three-year period. This percent reduction in hs-CRP associated with the AHEI-derived vegetable score is lower than the percent reduction in hs-CRP associated with the DASH-derived vegetable score. No significant associations were observed in the pooled analysis or T1D group.

## 4. Discussion

We observed a significant inverse association between blueberry consumption and serum hs-CRP level in the models adjusted for traditional cardiovascular risks in the non-DM group. Interestingly, this significant association was not maintained with the dietary pattern-derived fruit scores, including berries in the pooled or stratified analysis; this highlights the unique health benefits of these berry fruits versus other types of fruits, even when habitually consumed in lower amounts. When stratified by diabetes status, a significant inverse association between DASH-derived vegetable score and hs-CRP was observed in the non-DM group only. This significant inverse association in the non-DM group was also observed when vegetable intake was assessed using the AHEI pattern. These significant associations in the non-DM group may be related to the increased vegetable consumption within the three-year period. In the T1D group there was no association of fruit and/or vegetable scores with serum hs-CRP, suggesting that habitual consumption does not counteract the disease burden and its biomarkers.

Epidemiological studies have reported that increased DASH-derived dietary scores are associated with reduced CRP in the Cork and Kerry Diabetes and Heart Disease Study [59,60], a longitudinal study of a representative sample of the general population of Southern Ireland aged 50–69 years [61]. The baseline profile of this cohort included individuals with cardiovascular risk factors such as smoking, obesity, hypertension, physical inactivity, high cholesterol, and diabetes [61]. Serum CRP levels in these studies were determined using standard assays [59]. This sample was used in a cross-sectional analysis of DASH-derived dietary scores and CRP [59]. In this cross-sectional analysis, 8.5% of the individuals had type 2 diabetes, and the median CRP concentration was not elevated. A second cross-sectional analysis from the Cork and Kerry cohort, however, reported a 21.3% prevalence of type 2 diabetes [60]. This analysis categorized DASH-derived dietary scores in quartiles and also reported a significant inverse correlation between dietary scores and CRP, and significant differences in CRP levels between the lowest and highest quartiles of DASH scores [60]. These DASH-derived dietary scores were total scores, including all food groups.

Similarly, increased AHEI-derived dietary scores were negatively associated with hs-CRP in the CoLaus Study [24] and standard assay CRP in a U.S.-based observational study [62]. The cross-sectional analysis using data from the CoLaus Study had a sample with an average age of 57 years, and approximately 10% of the individuals had diabetes [24]. The U.S.-based study had a non-DM sample with an average age of 46 (males) and 44 (females) years with an average CRP level similar to our study at baseline and year three (not elevated) [62]. These associations were determined using AHEI-derived total dietary scores, including all food groups, fruits, and vegetables.

In these previous studies, the inverse associations were reported from DASH- and AHEI-derived scores inclusive of all dietary intake rather than solely fruits or vegetables. The aim of our current study was to determine adherence to fruits and vegetables specifically due to their anti-inflammatory and other cardioprotective properties. Our findings are similar to those of a cross-sectional observational study of U.S. adults that reported a significant inverse association between vegetables as a specific food group and CRP measured using a standard assay [63]. Using data from the National Health and Nutrition Examination Survey, individuals had elevated serum CRP levels, and there was a significant trend between increasing vegetable consumption (categorized in tertiles) and lower CRP levels [63]. Increasing vegetable consumption was also significantly associated with reduced odds for CRP greater than or equal to 3 mg/L among these individuals [63].

The inverse association between the DASH- and AHEI-derived vegetable scores and CRP (hs- and standard assay) is likely due to the known anti-inflammatory effects of vegetables [64,65]. Both of these dietary indices separate vegetables from legumes, so these scores represent intake of leafy green, cruciferous, root, stem, and bulb vegetables, which are rich in antioxidants and phytochemicals [66]. Phytochemicals found in cruciferous, leafy, and red-orange vegetables, such as isothiocyanates, carotenoids, and flavonoids, have been shown to reduce the production of inflammatory cytokines and inhibit nitric oxide production in cellular models [67,68,69,70]. In animal models, quercetin (a flavonoid) and lycopene (a carotenoid) significantly decreased standard assay CRP in insulin-resistant and type 2 diabetic rats, respectively [71,72]. It is likely that these anti-inflammatory effects of bioactive compounds in vegetables contribute to the associated reduction in CRP with habitual consumption, as observed in our human study. Interestingly, our inverse association between vegetable intake and hs-CRP was only observed in the non-DM controls. The non-DM controls are more similar to the primarily healthy populations in the previously mentioned epidemiological studies [24,59,62,63].

Specifically related to berries, the inverse association between blueberry intake and hs-CRP in the non-DM group is likely due to the fruit’s antioxidant properties. A number of anthocyanins are present in blueberries with a high antioxidant capacity that is second highest when compared to black currants, raspberries, red currants, and cranberries [73]. Blueberries have been shown to ameliorate oxidative stress biomarkers [74,75] and reduce hs-CRP levels [76] in animal models. It is likely that the increased antioxidant capacity and reduced oxidative stress contribute to the associated reduction in CRP. Our findings are similar to those of a cross-sectional analysis using a sample from the TwinsUK registry, which reported a significant inverse association between anthocyanin intake and hs-CRP levels in women [77]. Within this sample, 1.6% of the women were on diabetes or cholesterol-lowering drugs [77]. We did not find any significant association with total berry or strawberry intake in our pooled or stratified analysis. In randomized controlled trials reporting a reduction in hs-CRP, fresh and freeze-dried strawberries are provided at a dosage nearly 10 times the reported intake of our study [78]. The low dosage of strawberry intake may contribute to our insignificant findings, especially considering that blueberries have stronger antioxidant activity due to higher levels of anthocyanins [79].

Interestingly, the inverse associations of DASH- and AHEI-derived vegetable scores and blueberry intake with hs-CRP were not significant in our study for the T1D group. In our study, the median hs-CRP concentration of the T1D group was not categorically high; however, the T1D group had a higher systemic glycemic load as measured by hemoglobin A1c (HbA_1c_) compared to the non-DM group. In an observational study of non-institutionalized adults with type one and two diabetes, higher HbA_1c_ was significantly associated with higher CRP measured using a standard assay [80]. In subjects with an HbA1c between 9 and 10.9%, there was a significant 215% increase in odds of having an average CRP concentration greater than 0.30 mg/dL when adjusted for demographics, smoking, BMI, fasting insulin level, and length of time with diabetes [80]. In a cross-sectional analysis of adults with T1D, an average HbA_1c_ of 7.6% was significantly associated with higher hs-CRP compared to control groups with an average HbA1c of 5.3% [81]. The average HbA_1c_ concentrations in our study were similar to these levels across the three years. In studies reporting a negative association between dietary intake and hs-CRP in groups with T1D, the patients had an average HbA1c similar to our study of 7.6–8.6%, but the dietary patterns included all foods, not solely fruits and vegetables [45,46]. The hyperglycemia in T1D leads to other abnormalities, such as oxidative stress. In a cross-sectional analysis of women with type 2 diabetes, HbA_1c_ was negatively correlated to total antioxidant capacity and positively correlated with 8-iso-prostane, which are markers of oxidative stress [82]. While we did not measure oxidative stress in this study’s cohort, it is likely that the T1D group also has higher levels of oxidative stress and oxidation compared to the non-DM group. Thus, persistent hyperglycemia and related metabolic abnormalities, including oxidative stress in the T1D group, may have outweighed the cardioprotective effects of fruits and vegetables, and as a result, no protective associations were observed with hs-CRP in our study.

An additional factor that may have contributed to these insignificant findings in the T1D group is habitual fiber intake. A cross-sectional study of T1D patients with a relatively low average hs-CRP concentration reported that fiber intake greater than 30 g per day was inversely associated with hs-CRP [83]. In our study, the average intake of fiber was about 17 g. Our findings also contradict findings from a cross-sectional analysis of adherence to a semi-healthy diet and odds of high hs-CRP [46]. In this study, all patients had T1D, and 27.4% of the patients had high levels of hs-CRP [46]. The semi-healthy diet, however, included whole grains in its analysis and individuals in the highest tertile of adherence consumed approximately 30g of fiber per day [46]. The anti-inflammatory properties of fiber may have stronger cardioprotective effects in systemic hyperglycemia than the phytochemicals of fruits and vegetables.

Our findings in T1D adults also differ from previously reported decreased CRP levels after several years of healthy eating patterns in patients with type 2 diabetes [29,34]. These eating patterns often include other plant-based foods rich in antioxidants and phytochemicals with increased potential to modulate CRP levels. The purpose of our study, however, was to isolate the effects of fruit and vegetable consumption, which may have lower anti-inflammatory capacity when compared to full dietary intake of all plant-based foods. In addition, our insignificant findings may be related to the relatively low hs-CRP levels at the baseline of our participants. CRP levels in type 2 diabetes are often elevated, increasing the potential margin of reduction. Despite not finding significant reductions in hs-CRP in the T1D group, we did observe significant differences (reductions) in HbA1c over three years, which could be attributed to an overall healthy diet, including fruit and vegetable intake, in the T1D group. Our group has previously reported that improved glycemic control is associated with increased intake of dietary fiber, a component of fruits and vegetables, in adults with prediabetes in the same study [48]. In addition, we have reported an association between increased intake of dietary antioxidants and insulin sensitivity in T1D [84] and inverse associations between diet quality and cardiovascular disease risk in T1D adults [85]. These health-promoting effects of diets, though not reflected in hs-CRP in the current report, continue to support the role of healthy dietary patterns in T1D.

The strength of our study lies in its large sample size, including T1D and non-DM participants, providing a robust sample composition for inference. To our knowledge, this is the first study to assess the associations of dietary index-derived fruit and vegetable scores with hs-CRP in adults with and without diabetes in the same cohort. The dietary indices used in this study have many strengths, including common characteristics and generalizability across findings due to consistency in measurements [53,54,86]. Additionally, our analysis included data collected from two visits over three years. The longitudinal design also allowed us to detect temporal changes in circulating hs-CRP in adults with and without diabetes.

While our study has these methodological strengths, there are also a few limitations. The observational design does not allow for the determination of causation between dietary intake and circulating hs-CRP among the groups. In addition, using the food frequency questionnaire to collect dietary information introduces the potential for recall or social desirability bias. That said, the tool was administered in accordance with its validation. Finally, despite adjusting for all relevant covariates in our models, there is still the potential for residual confounding.

## 5. Conclusions

In conclusion, three-year data from the CACTI study show that blueberry consumption and vegetable intake, as measured using the DASH- and AHEI-derived dietary scores, are inversely associated with circulating hs-CRP in adults without diabetes. Thus, blueberry and vegetable intake should be emphasized in dietary recommendations to mitigate systemic inflammation and subsequent health outcomes related to elevated hs-CRP in the general population. In the T1D group, the lack of significant associations suggests that systemic hyperglycemia may negate the cardioprotective features of berries and vegetables. The lack of significant findings may also be related to the glycemic control and relatively low hs-CRP levels of our T1D participants. There is the possibility that T1D patients with poor glycemic control or higher hs-CRP than what we observed in our sample could benefit from a similar level of fruit and vegetable intake, but this would require future investigation. Future research should also examine interventions with increased amounts of fruits and vegetables that increase fiber dosage to alleviate inflammation in adults with T1D and prevent or delay micro- and macrovascular complications.

## Figures and Tables

**Table 1:** Baseline and year three characteristics of participants.

	Non-DM (*n* = 692)		Non-DM (*n* = 532)		Difference between Visits	T1D (*n* = 563)		T1D (*n* = 421)		Difference between Visits
	Baseline		Year Three (Visit 2)			Baseline		Year Three (Visit 2)		
**Variables**	**Count**	**%**	**Count**	**%**	***p*-Value**	**Count**	**%**	**Count**	**%**	***p*-Value**
Sex (Female)	349	50	265	51	0.9480	319	57	243	56	0.6180
Hispanic	59	9	41	8	0.6040	15	3	7	2	0.2931
Non-Hispanic White	582	84	461	88	0.2128	536	95	407	94	0.2535
Never Smoker	464	67	361	69	0.8407	383	68	296	68	0.4348
Current Smoker	59	9	46	9	0.9570	65	12	29	7	0.0140
Former Smoker	165	24	124	24	0.7975	114	20	95	22	0.3766
	**Median**	**IQR**	**Median**	**IQR**		**Median**	**IQR**	**Median**	**IQR**	
Hs-CRP (mg/dL)	1.2	(0.9–2.0)	1.3	(0.6–3.2)	0.9269 ^¶^	1.2	(0.9–2.2)	1.5	(0.7–3.8)	0.1630 ^¶^
Physical Activity	84	(0–300)	60	(0–270)	0.1404 ^¶^	45	(0–300)	40	(0–263)	0.3410 ^¶^
	**Mean**	**SD**	**Mean**	**SD**		**Mean**	**SD**	**Mean**	**SD**	
Age (year)	39	9	43	9	<0.0001	37	9	40	9	<0.0001
BMI (kg/m^2^)	26.2	5	26.6	5	<0.0001	26.2	4	26.3	4	0.0052
Calories (kcal/day)	1821	619	1758	622	0.0178	1768	613	1732	598	0.8247
HbA1c (%)	5.5	0.4	5.3	0.5	<0.0001	7.9	1.2	7.6	1.1	<0.0001
SBP (mm Hg)	114	12	110	12	<0.0001	117	14	112	13	<0.0001
Total Berries ^†^	0.24	0.96	0.25	0.82	0.4681	0.32	1.08	0.24	0.84	0.6002
Strawberries ^†^	0.22	0.68	0.20	0.47	0.8723	0.29	0.89	0.22	0.69	0.5839
Blueberries ^†^	0.24	0.84	0.23	0.67	0.6246	0.28	0.89	0.21	0.66	0.2901
Total Fruit *^†^	2.16	3.62	2.42	5.76	0.1289	2.61	4.18	2.26	4.66	0.4051
Total Vegetables *^†^	2.97	5.47	3.29	7.45	0.0415	3.84	8.97	3.07	2.98	0.1595
Dietary Fiber Intake	16.79	8.13	16.42	8.04	0.2457	16.72	7.80	15.75	7.88	0.0352
MSDPS Fruit Score	4.50	2.83	4.43	2.83	0.4830	4.56	2.94	4.50	2.82	0.2335
MSDPS Vegetable Score	3.83	2.27	3.88	2.72	0.3249	3.93	2.42	3.85	2.31	0.7111
DASH Fruit Score	2.89	1.41	2.98	1.40	0.0648	3.08	1.40	2.95	1.38	0.1274
DASH Vegetable Score	2.94	1.39	2.97	1.39	0.0971	3.07	1.43	2.99	1.42	0.8788
AHEI Fruit Score	3.08	2.65	3.27	2.68	0.0719	3.33	2.72	3.14	2.55	0.3902
AHEI Vegetable Score	4.79	2.86	4.85	2.79	0.0685	5.12	3.00	5.00	2.89	0.7806

Abbreviations: AHEI = Alternative Healthy Eating Index; BMI—Body Mass Index; CI = Confidence Interval; DASH = Dietary Approaches to Stop Hypertension; DM = Diabetes Mellitus; HbA1c = Glycated Hemoglobin; Hs-CRP = High-Sensitivity C-Reactive Protein; MSDPS = Mediterranean-Style Dietary Pattern Score; SBP = Systolic Blood Pressure; SD = Standard Deviation; T1D = Type I Diabetes. * Based on MSDPS and DASH total fruit and vegetable servings per day; ^†^ servings per day; ^¶^ Wilcoxon Rank Sum Test with Monte Carlo Estimation.

**Table 2:** The longitudinal associations between berry consumption and hs-CRP over three years.

Variables		Pooled		Non-DM		T1D	
		% Change (95% CI)	*p*-Value	% Change (95% CI)	*p*-Value	% Change (95% CI)	*p*-Value
Total Berries	Model 1	−1.56 (−4.53, 1.50)	0.3137	−2.61 (−6.64, 1.58)	0.2185	−0.44 (−4.75, 4.07)	0.8465
	Model 2	−1.48 (−4.39, 1.51)	0.3281	−3.43 (−7.16, 0.45)	0.0825	0.78 (−3.73, 5.51)	0.7385
Strawberry	Model 1	−1.67 (−6.28, 3.16)	0.4906	−2.29 (−8.84, 4.74)	0.5134	−1.18 (−7.62, 5.71)	0.7291
	Model 2	−1.66 (−6.31, 3.22)	0.4976	−4.73 (−10.83, 1.78)	0.1506	0.86 (−6.22, 8.47)	0.8173
Blueberry	Model 1	−5.09 (−10.51, 0.65)	0.0809	−6.50 (−13.38, 0.92)	0.0844	−3.28 (−11.7, 5.93)	0.4710
	Model 2	−4.80 (−9.95, 0.65)	0.0830	**−8.29 (−14.50, −1.64)**	**0.0155**	−0.15 (−8.68, 9.18)	0.9733

Model 1: Age + Sex + Calories + Visit + Diabetes Status (for pooled analyses); Model 2: Model 1 + Systolic Blood Pressure + Body Mass Index + Smoking Status + Patient Illness Status; Bold ≤ 0.05. Abbreviations: CI = Confidence Interval; DM = Diabetes Mellitus; T1D = Type I Diabetes.

**Table 3:** The longitudinal associations between berry consumption and odds of elevated hs-CRP (greater than 3 mg/dL) over three years.

Variables		Pooled		Non-DM		T1D	
		OR (95% CI)	*p*-Value	OR (95% CI)	*p*-Value	OR (95% CI)	*p*-Value
Total Berries	Model 1	0.91 (0.75, 1.06)	0.2518	0.87 (0.59, 1.10)	0.3039	0.94 (0.72, 1.12)	0.5281
	Model 2	0.95 (0.84, 1.09)	0.4758	0.92 (0.76, 1.12)	0.4042	1.01 (0.83, 1.23)	0.9487
Strawberry	Model 1	0.88 (0.61, 1.10)	0.3160	0.82 (0.23, 1.21)	0.4159	0.91 (0.59, 1.16)	0.5371
	Model 2	0.93 (0.76, 1.15)	0.5027	0.86 (0.62, 1.19)	0.3622	1.01 (0.76, 1.33)	0.9732
Blueberry	Model 1	0.89 (0.55, 1.14)	0.4161	0.83 (0.25, 1.22)	0.4370	0.93 (0.45, 1.26)	0.7166
	Model 2	0.94 (0.73, 1.20)	0.5998	0.88 (0.62, 1.24)	0.4632	1.02 (0.66, 1.57)	0.9426

Model 1: Age + Sex + Calories + Visit + Diabetes Status (for pooled analyses); Model 2: Model 1 + Systolic Blood Pressure + Body Mass Index + Smoking Status + Patient Illness Status; Bold ≤ 0.05. Abbreviations: CI = Confidence Interval; DM = Diabetes Mellitus; OR = Odds Ratio; T1D = Type I Diabetes.

**Table 4:** Fruit and vegetable scores and their association with log-transformed hs-CRP over three years.

Dietary Pattern Scores	Variables	Models	Pooled			Non-DM			T1D		
			%Change	95% CI	*p*-Value	%Change	95% CI	*p*-Value	%Change	95% CI	*p*-Value
MSDPS	Fruit Score (including berries)	Model 1	−0.68	(−1.75, 0.41)	0.2232	−1.23	(−2.62, 0.18)	0.0867	0.12	(−1.55, 1.83)	0.8862
		Model 2	0.13	(−0.95, 1.22)	0.8180	0.08	(−1.27, 1.45)	0.9057	0.27	(−1.45, 2.02)	0.7630
	Fruit Score (excluding berries)	Model 1	−0.78	(−1.91, 0.36)	0.1770	−1.11	(−2.56, 0.36)	0.1374	−0.38	(−2.14, 1.41)	0.6778
		Model 2	−0.01	(−1.13, 1.13)	0.9854	0.11	(−1.29, 1.54)	0.8737	−0.22	(−2.01, 1.60)	0.8139
	Vegetable Score	Model 1	−0.63	(−2.04, 0.80)	0.3846	−1.78	(−3.64, 0.12)	0.0657	0.45	(−1.65, 2.59)	0.6756
		Model 2	−0.59	(−1.97, 0.81)	0.4067	−1.29	(−3.07, 0.52)	0.1623	0.03	(−2.08, 2.19)	0.9756
DASH	Fruit Score (including berries)	Model 1	−1.59	(−4.00, 0.88)	0.2050	−2.07	(−5.18, 1.15)	0.2052	−1.38	(−5.08, 2.48)	0.4780
		Model 2	−0.77	(−3.15, 1.67)	0.5335	−0.62	(−3.62, 2.48)	0.6911	−1.10	(−4.83, 2.77)	0.5716
	Fruit Score (excluding berries)	Model 1	−1.67	(−4.07, 0.79)	0.1813	−1.56	(−4.66, 1.64)	0.3340	−2.21	(−5.89, 1.62)	0.2546
		Model 2	−0.86	(−3.23, 1.57)	0.4833	−0.14	(−3.14, 2.95)	0.9287	−1.92	(−5.61, 1.92)	0.3220
	Vegetable Score	Model 1	−1.99	(−4.35, 0.43)	0.1057	**−3.69**	**(−6.69, −0.59)**	**0.0202**	0.08	(−3.64, 3.95)	0.9655
		Model 2	−2.05	(−4.35, 0.31)	0.0879	**−3.22**	**(−6.08, −0.28)**	**0.0321**	−0.47	(−4.18, 3.39)	0.8090
AHEI	Fruit Score (including berries)	Model 1	−0.64	(−1.89, 0.62)	0.3192	−0.95	(−2.60, 0.72)	0.2612	−0.39	(−2.28, 1.54)	0.6896
		Model 2	−0.24	(−1.47, 1.00)	0.7002	−0.47	(−2.04, 1.14)	0.5669	−0.12	(−2.03, 1.81)	0.8985
	Fruit Score (excluding berries)	Model 1	−0.66	(−2.00, 0.71)	0.3426	−0.91	(−2.66, 0.88)	0.3189	−0.50	(−2.55, 1.60)	0.6391
		Model 2	−0.25	(−1.57, 1.08)	0.7107	−0.36	(−2.05, 1.35)	0.6746	−0.27	(−2.31, 1.82)	0.7989
	Vegetable Score	Model 1	−0.83	(−1.99, 0.34)	0.1630	**−1.53**	**(−3.05, 0.02)**	**0.0525**	−0.14	(−1.91, 1.66)	0.8794
		Model 2	−0.99	(−2.12, 0.15)	0.0897	**−1.45**	**(−2.88, 0.00)**	**0.0504**	−0.50	(−2.27, 1.31)	0.5857

Model 1: Age + Sex + Calories + Visit + Diabetes Status (for pooled analyses); Model 2: Model 1 + Systolic Blood Pressure + Body Mass Index + Smoking Status + Patient Illness Status; Bold ≤ 0.05. Abbreviations: AHEI = Alternative Healthy Eating Index; CI = Confidence Interval; DASH = Dietary Approaches to Stop Hypertension; DM = Diabetes Mellitus; MSDPS = Mediterranean-Style Dietary Pattern Score; T1D = Type I diabetes.

## Data Availability

The data are not publicly available due to patient privacy.

## References

[1] Noncommunicable Diseases. https://www.who.int/news-room/fact-sheets/detail/noncommunicable-diseases.

[2] Vaduganathan M., Mensah G.A., Turco J.V., Fuster V., Roth G.A. (2022). The Global Burden of Cardiovascular Diseases and Risk. J. Am. Coll. Cardiol..

[3] GBD 2019 Chronic Respiratory Diseases Collaborators (2023). Global Burden of Chronic Respiratory Diseases and Risk Factors, 1990–2019: An Update from the Global Burden of Disease Study 2019. eClinicalMedicine.

[4] GBD 2021 Diabetes Collaborators (2023). Global, Regional, and National Burden of Diabetes from 1990 to 2021, with Projections of Prevalence to 2050: A Systematic Analysis for the Global Burden of Disease Study 2021. Lancet.

[5] Furman D., Campisi J., Verdin E., Carrera-Bastos P., Targ S., Franceschi C., Ferrucci L., Gilroy D.W., Fasano A., Miller G.W. (2019). Chronic Inflammation in the Etiology of Disease across the Life Span. Nat. Med..

[6] Cai X., Li J., Cai W., Chen C., Ma J., Xie Z., Dong Y., Liu C., Xue R., Zhao J. (2021). Meta-Analysis of Type 1 Diabetes Mellitus and Risk of Cardiovascular Disease. J. Diabetes Complicat..

[7] Cano-Cano F., Gómez-Jaramillo L., Ramos-García P., Arroba A.I., Aguilar-Diosdado M. (2022). IL-1β Implications in Type 1 Diabetes Mellitus Progression: Systematic Review and Meta-Analysis. J. Clin. Med..

[8] Chen Y.-L., Qiao Y.-C., Pan Y.-H., Xu Y., Huang Y.-C., Wang Y.-H., Geng L.-J., Zhao H.-L., Zhang X.-X. (2017). Correlation between Serum Interleukin-6 Level and Type 1 Diabetes Mellitus: A Systematic Review and Meta-Analysis. Cytokine.

[9] Qiao Y., Chen Y., Pan Y., Tian F., Xu Y., Zhang X., Zhao H. (2017). The Change of Serum Tumor Necrosis Factor Alpha in Patients with Type 1 Diabetes Mellitus: A Systematic Review and Meta-Analysis. PLoS ONE.

[10] Eizirik D.L., Colli M.L., Ortis F. (2009). The Role of Inflammation in Insulitis and Beta-Cell Loss in Type 1 Diabetes. Nat. Rev. Endocrinol..

[11] Pirot P., Eizirik D.L., Cardozo A.K. (2006). Interferon-Gamma Potentiates Endoplasmic Reticulum Stress-Induced Death by Reducing Pancreatic Beta Cell Defence Mechanisms. Diabetologia.

[12] Navarro J.F., Mora C. (2005). Role of Inflammation in Diabetic Complications. Nephrol. Dial. Transplant..

[13] Schalkwijk C.G., Poland D.C., van Dijk W., Kok A., Emeis J.J., Dräger A.M., Doni A., van Hinsbergh V.W., Stehouwer C.D. (1999). Plasma Concentration of C-Reactive Protein Is Increased in Type I Diabetic Patients without Clinical Macroangiopathy and Correlates with Markers of Endothelial Dysfunction: Evidence for Chronic Inflammation. Diabetologia.

[14] Akinboboye O., Williams J.S., Garacci E., Egede L.E. (2022). The Relationship between C-Reactive Protein and Mortality in Adults with Diabetes: Influences of Demographic Characteristics, Lifestyle Behaviors, and Medications. Nutr. Metab. Cardiovasc. Dis..

[15] Tian R., Tian M., Wang L., Qian H., Zhang S., Pang H., Liu Z., Fang L., Shen Z. (2019). C-Reactive Protein for Predicting Cardiovascular and All-Cause Mortality in Type 2 Diabetic Patients: A Meta-Analysis. Cytokine.

[16] Romero-Cabrera J.L., Ankeny J., Fernández-Montero A., Kales S.N., Smith D.L. (2022). A Systematic Review and Meta-Analysis of Advanced Biomarkers for Predicting Incident Cardiovascular Disease among Asymptomatic Middle-Aged Adults. Int. J. Mol. Sci..

[17] Best L.G., Zhang Y., Lee E.T., Yeh J.-L., Cowan L., Palmieri V., Roman M., Devereux R.B., Fabsitz R.R., Tracy R.P. (2005). C-Reactive Protein as a Predictor of Cardiovascular Risk in a Population With a High Prevalence of Diabetes. Circulation.

[18] Zhang D., Sun M., Samols D., Kushner I. (1996). STAT3 Participates in Transcriptional Activation of the C-Reactive Protein Gene by Interleukin-6 (∗). J. Biol. Chem..

[19] de Almeida Júnior R.F., de Souza K.S.C., Galdino O.A., da Silva Junior A.A., Arrais R.F., Machado P.R.L., Farias K.J.S., de Rezende A.A. (2020). Chloroquine as a Promising Adjuvant Therapy for Type 1 Diabetes Mellitus. Sci. Rep..

[20] Pollack R.M., Donath M.Y., LeRoith D., Leibowitz G. (2016). Anti-Inflammatory Agents in the Treatment of Diabetes and Its Vascular Complications. Diabetes Care.

[21] Maithili Karpaga Selvi N., Sridhar M.G., Swaminathan R.P., Sripradha R. (2015). Efficacy of Turmeric as Adjuvant Therapy in Type 2 Diabetic Patients. Indian. J. Clin. Biochem..

[22] Moslemi E., Musazadeh V., Kavyani Z., Naghsh N., Shoura S.M.S., Dehghan P. (2022). Efficacy of Vitamin D Supplementation as an Adjunct Therapy for Improving Inflammatory and Oxidative Stress Biomarkers: An Umbrella Meta-Analysis. Pharmacol. Res..

[23] Zwickey H., Horgan A., Hanes D., Schiffke H., Moore A., Wahbeh H., Jordan J., Ojeda L., McMurry M., Elmer P. (2019). Effect of the Anti-Inflammatory Diet in People with Diabetes and Pre-Diabetes: A Randomized Controlled Feeding Study. J. Restor. Med..

[24] Piccand E., Vollenweider P., Guessous I., Marques-Vidal P. (2019). Association between Dietary Intake and Inflammatory Markers: Results from the CoLaus Study. Public Health Nutr..

[25] Gao X., Bermudez O.I., Tucker K.L. (2004). Plasma C-Reactive Protein and Homocysteine Concentrations Are Related to Frequent Fruit and Vegetable Intake in Hispanic and Non-Hispanic White Elders. J. Nutr..

[26] Oliveira A., Rodríguez-Artalejo F., Lopes C. (2009). The Association of Fruits, Vegetables, Antioxidant Vitamins and Fibre Intake with High-Sensitivity C-Reactive Protein: Sex and Body Mass Index Interactions. Eur. J. Clin. Nutr..

[27] Nanri H., Nakamura K., Hara M., Higaki Y., Imaizumi T., Taguchi N., Sakamoto T., Horita M., Shinchi K., Tanaka K. (2011). Association between Dietary Pattern and Serum C-Reactive Protein in Japanese Men and Women. J. Epidemiol..

[28] Haghighatdoost F., Bellissimo N., Totosy de Zepetnek J.O., Rouhani M.H. (2017). Association of Vegetarian Diet with Inflammatory Biomarkers: A Systematic Review and Meta-Analysis of Observational Studies. Public Health Nutr..

[29] Jacobs S., Boushey C.J., Franke A.A., Shvetsov Y.B., Monroe K.R., Haiman C.A., Kolonel L.N., Le Marchand L., Maskarinec G. (2017). A Priori-Defined Diet Quality Indices, Biomarkers and Risk for Type 2 Diabetes in Five Ethnic Groups: The Multiethnic Cohort. Br. J. Nutr..

[30] Bhupathiraju S.N., Tucker K.L. (2011). Greater Variety in Fruit and Vegetable Intake Is Associated with Lower Inflammation in Puerto Rican Adults. Am. J. Clin. Nutr..

[31] Julia C., Meunier N., Touvier M., Ahluwalia N., Sapin V., Papet I., Cano N., Hercberg S., Galan P., Kesse-Guyot E. (2013). Dietary Patterns and Risk of Elevated C-Reactive Protein Concentrations 12 Years Later. Br. J. Nutr..

[32] Nouri F., Sadeghi M., Mohammadifard N., Roohafza H., Feizi A., Sarrafzadegan N. (2021). Longitudinal Association between an Overall Diet Quality Index and Latent Profiles of Cardiovascular Risk Factors: Results from a Population Based 13-Year Follow up Cohort Study. Nutr. Metab..

[33] Hosseini B., Berthon B.S., Saedisomeolia A., Starkey M.R., Collison A., Wark P.A.B., Wood L.G. (2018). Effects of Fruit and Vegetable Consumption on Inflammatory Biomarkers and Immune Cell Populations: A Systematic Literature Review and Meta-Analysis. Am. J. Clin. Nutr..

[34] Sánchez-Rosales A.I., Guadarrama-López A.L., Gaona-Valle L.S., Martínez-Carrillo B.E., Valdés-Ramos R. (2022). The Effect of Dietary Patterns on Inflammatory Biomarkers in Adults with Type 2 Diabetes Mellitus: A Systematic Review and Meta-Analysis of Randomized Controlled Trials. Nutrients.

[35] Koelman L., Egea Rodrigues C., Aleksandrova K. (2022). Effects of Dietary Patterns on Biomarkers of Inflammation and Immune Responses: A Systematic Review and Meta-Analysis of Randomized Controlled Trials. Adv. Nutr..

[36] Neale E.P., Batterham M.J., Tapsell L.C. (2016). Consumption of a Healthy Dietary Pattern Results in Significant Reductions in C-Reactive Protein Levels in Adults: A Meta-Analysis. Nutr. Res..

[37] Sarkhosh-Khorasani S., Hosseinzadeh M. (2021). The Effect of Grape Products Containing Polyphenols on C-Reactive Protein Levels: A Systematic Review and Meta-Analysis of Randomised Controlled Trials. Br. J. Nutr..

[38] Luís Â., Domingues F., Pereira L. (2018). Association between Berries Intake and Cardiovascular Diseases Risk Factors: A Systematic Review with Meta-Analysis and Trial Sequential Analysis of Randomized Controlled Trials. Food Funct..

[39] Schell J., Betts N.M., Lyons T.J., Basu A. (2019). Raspberries Improve Postprandial Glucose and Acute and Chronic Inflammation in Adults with Type 2 Diabetes. Ann. Nutr. Metab..

[40] Soltani S., Chitsazi M.J., Salehi-Abargouei A. (2018). The Effect of Dietary Approaches to Stop Hypertension (DASH) on Serum Inflammatory Markers: A Systematic Review and Meta-Analysis of Randomized Trials. Clin. Nutr..

[41] Hart M.J., Torres S.J., McNaughton S.A., Milte C.M. (2021). Dietary Patterns and Associations with Biomarkers of Inflammation in Adults: A Systematic Review of Observational Studies. Nutr. J..

[42] Keys A., Mienotti A., Karvonen M., Aravanis C., Blackburn H., Buzina R., Djordjevic B., Dontas A., Fidanza F., Keys M. (1986). The Diet and 15-Year Death Rate in the Seven Countries Study. Am. J. Epidemiol..

[43] George S.M., Ballard-Barbash R., Manson J.E., Reedy J., Shikany J.M., Subar A.F., Tinker L.F., Vitolins M., Neuhouser M.L. (2014). Comparing Indices of Diet Quality With Chronic Disease Mortality Risk in Postmenopausal Women in the Women’s Health Initiative Observational Study: Evidence to Inform National Dietary Guidance. Am. J. Epidemiol..

[44] van Bussel B.C.T., Soedamah-Muthu S.S., Henry R.M.A., Schalkwijk C.G., Ferreira I., Chaturvedi N., Toeller M., Fuller J.H., Stehouwer C.D.A. (2013). EURODIAB Prospective Complications Study Group Unhealthy Dietary Patterns Associated with Inflammation and Endothelial Dysfunction in Type 1 Diabetes: The EURODIAB Study. Nutr. Metab. Cardiovasc. Dis..

[45] Ahola A.J., Saraheimo M., Freese R., Forsblom C., Mäkimattila S., Groop P.-H. (2017). Association between Adherence to Dietary Recommendations and High-Sensitivity C-Reactive Protein Level in Type 1 Diabetes. Diabetes Res. Clin. Pract..

[46] Ebrahimi Z., Shojaeian Z., Amiri F., Esmaillzadeh A., Sadeghi O., Esteghamati A., Jahed S.A., Sedaghat S. (2023). Association of Major Dietary Patterns with Advanced Glycation End Products and High-Sensitivity C-Reactive Protein in People with Type 1 Diabetes Mellitus. Nutr. J..

[47] Dabelea D., Kinney G., Snell-Bergeon J.K., Hokanson J.E., Eckel R.H., Ehrlich J., Garg S., Hamman R.F., Rewers M. (2003). Effect of Type 1 Diabetes on the Gender Difference in Coronary Artery Calcification: A Role for Insulin Resistance? The Coronary Artery Calcification in Type 1 Diabetes (CACTI) Study. Diabetes.

[48] Basu A., Alman A.C., Snell-Bergeon J.K. (2019). Dietary Fiber Intake and Glycemic Control: Coronary Artery Calcification in Type 1 Diabetes (CACTI) Study. Nutr. J..

[49] Willett W.C., Sampson L., Browne M.L., Stampfer M.J., Rosner B., Hennekens C.H., Speizer F.E. (1988). The Use of a Self-Administered Questionnaire to Assess Diet Four Years in the Past. Am. J. Epidemiol..

[50] Willett W.C., Sampson L., Stampfer M.J., Rosner B., Bain C., Witschi J., Hennekens C.H., Speizer F.E. (1985). Reproducibility and Validity of a Semiquantitative Food Frequency Questionnaire. Am. J. Epidemiol..

[51] Hu F.B., Rimm E., Smith-Warner S.A., Feskanich D., Stampfer M.J., Ascherio A., Sampson L., Willett W.C. (1999). Reproducibility and Validity of Dietary Patterns Assessed with a Food-Frequency Questionnaire. Am. J. Clin. Nutr..

[52] Wirfält A.K., Jeffery R.W., Elmer P.J. (1998). Comparison of Food Frequency Questionnaires: The Reduced Block and Willett Questionnaires Differ in Ranking on Nutrient Intakes. Am. J. Epidemiol..

[53] Rumawas M.E., Dwyer J.T., Mckeown N.M., Meigs J.B., Rogers G., Jacques P.F. (2009). The Development of the Mediterranean-Style Dietary Pattern Score and Its Application to the American Diet in the Framingham Offspring Cohort. J. Nutr..

[54] Fung T.T., Chiuve S.E., McCullough M.L., Rexrode K.M., Logroscino G., Hu F.B. (2008). Adherence to a DASH-Style Diet and Risk of Coronary Heart Disease and Stroke in Women. Arch. Intern. Med..

[55] Chiuve S.E., Fung T.T., Rimm E.B., Hu F.B., McCullough M.L., Wang M., Stampfer M.J., Willett W.C. (2012). Alternative Dietary Indices Both Strongly Predict Risk of Chronic Disease. J. Nutr..

[56] Bjornstad P., Pyle L., Kinney G.L., Rewers M., Johnson R.J., Maahs D.M., Snell-Bergeon J.K. (2017). Adiponectin Is Associated with Early Diabetic Kidney Disease in Adults with Type 1 Diabetes: A Coronary Artery Calcification in Type 1 Diabetes (CACTI) Study. J. Diabetes Complicat..

[57] Ridker P.M. (2005). C-Reactive Protein, Inflammation, and Cardiovascular Disease: Clinical Update. Tex. Heart Inst. J..

[58] Falsey A.R., Walsh E.E., Francis C.W., Looney R.J., Kolassa J.E., Hall W.J., Abraham G.N. (2001). Response of C-Reactive Protein and Serum Amyloid A to Influenza A Infection in Older Adults. J. Infect. Dis..

[59] Millar S.R., Navarro P., Harrington J.M., Shivappa N., Hébert J.R., Perry I.J., Phillips C.M. (2022). Dietary Score Associations with Markers of Chronic Low-Grade Inflammation: A Cross-Sectional Comparative Analysis of a Middle- to Older-Aged Population. Eur. J. Nutr..

[60] Phillips C.M., Harrington J.M., Perry I.J. (2019). Relationship between Dietary Quality, Determined by DASH Score, and Cardiometabolic Health Biomarkers: A Cross-Sectional Analysis in Adults. Clin. Nutr..

[61] Kearney P.M., Harrington J.M., Mc Carthy V.J., Fitzgerald A.P., Perry I.J. (2013). Cohort Profile: The Cork and Kerry Diabetes and Heart Disease Study. Int. J. Epidemiol..

[62] Park K.H., Zaichenko L., Peter P., Davis C.R., Crowell J.A., Mantzoros C.S. (2014). Diet Quality Is Associated with Circulating C-Reactive Protein but Not Irisin Levels in Humans. Metabolism.

[63] Chun O.K., Chung S.-J., Claycombe K.J., Song W.O. (2008). Serum C-Reactive Protein Concentrations Are Inversely Associated with Dietary Flavonoid Intake in U.S. Adults. J. Nutr..

[64] Zhu F., Du B., Xu B. (2018). Anti-Inflammatory Effects of Phytochemicals from Fruits, Vegetables, and Food Legumes: A Review. Crit. Rev. Food Sci. Nutr..

[65] Roberts J.L., Moreau R. (2016). Functional Properties of Spinach (*Spinacia Oleracea* L.) Phytochemicals and Bioactives. Food Funct..

[66] Kumar A., P N., Kumar M., Jose A., Tomer V., Oz E., Proestos C., Zeng M., Elobeid T., K S. (2023). Major Phytochemicals: Recent Advances in Health Benefits and Extraction Method. Molecules.

[67] Jung Y.J., Jung J.I., Cho H.J., Choi M.-S., Sung M.-K., Yu R., Kang Y.-H., Park J.H.Y. (2014). Berteroin Present in Cruciferous Vegetables Exerts Potent Anti-Inflammatory Properties in Murine Macrophages and Mouse Skin. Int. J. Mol. Sci..

[68] Kim J.E., Clark R.M., Park Y., Lee J., Fernandez M.L. (2012). Lutein Decreases Oxidative Stress and Inflammation in Liver and Eyes of Guinea Pigs Fed a Hypercholesterolemic Diet. Nutr. Res. Pract..

[69] Hadad N., Levy R. (2012). The Synergistic Anti-Inflammatory Effects of Lycopene, Lutein, β-Carotene, and Carnosic Acid Combinations via Redox-Based Inhibition of NF-κB Signaling. Free Radic. Biol. Med..

[70] Kim H.K., Cheon B.S., Kim Y.H., Kim S.Y., Kim H.P. (1999). Effects of Naturally Occurring Flavonoids on Nitric Oxide Production in the Macrophage Cell Line RAW 264.7 and Their Structure–Activity Relationships. Biochem. Pharmacol..

[71] Mahmoud M.F., Hassan N.A., El Bassossy H.M., Fahmy A. (2013). Quercetin Protects against Diabetes-Induced Exaggerated Vasoconstriction in Rats: Effect on Low Grade Inflammation. PLoS ONE.

[72] Zheng Z., Yin Y., Lu R., Jiang Z. (2019). Lycopene Ameliorated Oxidative Stress and Inflammation in Type 2 Diabetic Rats. J. Food Sci..

[73] Borges G., Degeneve A., Mullen W., Crozier A. (2010). Identification of Flavonoid and Phenolic Antioxidants in Black Currants, Blueberries, Raspberries, Red Currants, and Cranberries. J. Agric. Food Chem..

[74] Lee S., Keirsey K.I., Kirkland R., Grunewald Z.I., Fischer J.G., de La Serre C.B. (2018). Blueberry Supplementation Influences the Gut Microbiota, Inflammation, and Insulin Resistance in High-Fat-Diet–Fed Rats. J. Nutr..

[75] Elks C.M., Reed S.D., Mariappan N., Shukitt-Hale B., Joseph J.A., Ingram D.K., Francis J. (2011). A Blueberry-Enriched Diet Attenuates Nephropathy in a Rat Model of Hypertension via Reduction in Oxidative Stress. PLoS ONE.

[76] Vendrame S., Daugherty A., Kristo A.S., Riso P., Klimis-Zacas D. (2013). Wild Blueberry (Vaccinium Angustifolium) Consumption Improves Inflammatory Status in the Obese Zucker Rat Model of the Metabolic Syndrome. J. Nutr. Biochem..

[77] Jennings A., Welch A.A., Spector T., Macgregor A., Cassidy A. (2014). Intakes of Anthocyanins and Flavones Are Associated with Biomarkers of Insulin Resistance and Inflammation in Women1, 2. J. Nutr..

[78] Gao Q., Qin L.-Q., Arafa A., Eshak E.S., Dong J.-Y. (2020). Effects of Strawberry Intervention on Cardiovascular Risk Factors: A Meta-Analysis of Randomised Controlled Trials. Br. J. Nutr..

[79] Huang W., Zhang H., Liu W., Li C. (2012). Survey of Antioxidant Capacity and Phenolic Composition of Blueberry, Blackberry, and Strawberry in Nanjing. J. Zhejiang Univ. Sci. B.

[80] King D.E., Mainous A.G., Buchanan T.A., Pearson W.S. (2003). C-Reactive Protein and Glycemic Control in Adults with Diabetes. Diabetes Care.

[81] Gautam D., Adhikari S., Thapa R., Kharel L. (2023). Study to Determine between HbA1C and C-Reactive Protein in Diabetes Mellitus. J. Pathol. Nepal..

[82] Khosrowbeygi A., Gholami M., Zarei P., Sedeh B.S., Rezvanfar M.R. (2019). Correlations between Biomarkers of Oxidative Stress, Glycemic Control and Insulin Resistance in Women with Type 2 Diabetes. Clin. Diabetol..

[83] Bernaud F.S.R., Beretta M.V., do Nascimento C., Escobar F., Gross J.L., Azevedo M.J., Rodrigues T.C. (2014). Fiber Intake and Inflammation in Type 1 Diabetes. Diabetol. Metab. Syndr..

[84] Basu A., Alman A.C., Snell-Bergeon J.K. (2022). Associations of Dietary Antioxidants with Glycated Hemoglobin and Insulin Sensitivity in Adults with and without Type 1 Diabetes. J. Diabetes Res..

[85] Richardson L.A., Basu A., Chien L.-C., Pang T., Alman A.C., Snell-Bergeon J.K. (2024). Longitudinal Associations of the Alternative Healthy Eating Index with Coronary Artery Calcification and Pericardial Adiposity in US Adults with and without Type 1 Diabetes. Nutr. Metab. Cardiovasc. Dis..

[86] McCullough M.L., Feskanich D., Stampfer M.J., Giovannucci E.L., Rimm E.B., Hu F.B., Spiegelman D., Hunter D.J., Colditz G.A., Willett W.C. (2002). Diet Quality and Major Chronic Disease Risk in Men and Women: Moving toward Improved Dietary Guidance. Am. J. Clin. Nutr..

